# Efficacy of a Novel PCV2d and *Mycoplasma hyopneumoniae* Combined Vaccine in Piglets with High and Low Levels of PCV2 Maternally Derived Antibodies at Vaccination

**DOI:** 10.3390/vaccines13101076

**Published:** 2025-10-21

**Authors:** Mònica Sagrera, Laura Garza-Moreno, Àlex Cobos, Anna Maria Llorens, Eva Huerta, Mónica Pérez, Diego Pérez, David Espigares, Joaquim Segalés, Marina Sibila

**Affiliations:** 1 IRTA, Animal Health, Centre de Recerca en Sanitat Animal (CReSA), Campus de la Universitat Autònoma de Barcelona (UAB), 08193 Bellaterra, Catalonia, Spain; monica.sagrera@irta.cat (M.S.);; 2 Unitat mixta d’investigació IRTA-UAB en Sanitat Animal, Centre de Recerca en Sanitat Animal (CReSA), Campus de la Universitat Autònoma de Barcelona (UAB), 08193 Bellaterra, Catalonia, Spain; 3 Ceva Salud Animal, Avenida Diagonal, 08028 Barcelona, Spain; laura.garza@ceva.com (L.G.-M.);; 4 WOAH Collaborating Center for Research and Control of Emerging and Re-emerging Pig Diseases (IRTA-CReSA), 08193 Bellaterra, Barcelona, Spain; 5 Departament de Sanitat i Anatomia Animals, Facultat de Veterinària, Universitat Autònoma de Barcelona (UAB), 08193 Bellaterra, Barcelona, Spain

**Keywords:** porcine circovirus 2, maternally derived antibodies, vaccine, cytokines, quantitative PCR, in situ hybridisation

## Abstract

**Background/Objectives:** Maternally derived antibody (MDA) levels of porcine circovirus 2 (PCV2) may eventually interfere with humoral response and vaccination efficacy. This study aimed to evaluate the efficacy of a ready-to-use PCV2d and *Mycoplasma hyopneumoniae* combined vaccine in piglets with different PCV2 MDA levels at vaccination in an experimental inoculation with a heterologous viral genotype. **Methods:** Forty-eight piglets were allocated into vaccinated (V) and non-vaccinated (NV) groups with high (H) and low (L) PCV2 MDA subgroups (H-V, H-NV, L-V, L-NV). At 3 weeks of age, the piglets received either one dose of vaccine or placebo. Five weeks later, all animals were intranasally challenged with a PCV2b inoculum. Body weight was registered at different time points. Blood samples, peripheral blood mononuclear cells and tracheobronchial lymph nodes (TBLN) were collected and used to assess viraemia, viral load, humoral and cellular responses and histological lesions. **Results:** The V group showed higher PCV2 antibody levels from challenge onwards, along with a lower percentage of viraemic pigs and reduced viral load in serum at 2 and 3 weeks post-challenge (wpc) and in TBLN tissues compared to the NV group. The H-V group had the highest antibody levels post-challenge, showed no detectable viraemia and had a lower overall amount of virus in tissues. The NV group (especially H-NV) exhibited increased levels of IFN-γ, IFN-α and TNF-α post-challenge. **Conclusions:** The tested vaccine elicited humoral and cellular immune responses and reduced viral presence in serum and tissues, demonstrating efficacy in a PCV2 subclinical infection model despite high MDA levels at the time of vaccination. Understanding both humoral and cellular immune responses according to different MDA levels can help design more effective vaccination strategies against PCV2.

## 1. Introduction

Porcine circovirus 2 (PCV2) is one of the major pathogens in the swine industry, causing significant economic losses worldwide [1,2,3]. This virus is the causative agent of porcine circovirus diseases (PCVDs). The PCV2 subclinical infection (PCV2-SI) is the most common outcome nowadays [4], causing a potential reduction of 10 to 40 g in the average daily weight gain (ADWG) [5,6]. PCV2 systemic disease, PCV2 reproductive disease and porcine dermatitis and nephropathy syndrome are infrequent nowadays due to the systematic use of PCV2 vaccines worldwide [6,7]. In fact, vaccination against this virus has been widely adopted as a primary tool due to its effectiveness in controlling PCVDs and improving production parameters [6]. PCV2 vaccination not only reduces clinical signs and lesions associated with the infection but also decreases the percentage of viraemic pigs, the viral load and the transmission within herds [7].

Nine PCV2 genotypes have been described so far [7,8,9], including PCV2a, PCV2b and PCV2d, globally distributed and considered the most prevalent ones [8]. Currently, PCV2d has become the most frequently found genotype in many European countries [8,10,11,12], as well as in United States of America and China [13,14,15,16]. Although most of the PCV2 vaccines available in the market since 2008 are derived from the PCV2a genotype and consist of the capsid protein encoded by ORF2, cross-protection with PCV2b and PCV2d has been demonstrated [17,18,19]. PCV2 vaccines combining more than one genotype have been licensed recently in the international market [20]. Additionally, there are ready-to-mix and ready-to-use combined vaccines with *Mycoplasma (M.) hyopneumoniae* based on different technologies, such as PCV2 inactivated chimeric recombinant vaccines expressing ORF2 of PCV2a and PCV2b [21] and subunit vaccines based on PCV2a ORF-2 [22].

Maternally derived antibodies (MDAs) are crucial to protect the piglet at an early age, before it generates its own immune response following vaccination [7,23]. However, when PCV2 MDA levels are relatively high or very high at the moment of vaccination, they may interfere with vaccine-induced seroconversion [24,25,26,27]. Importantly, this negative effect of MDAs on the humoral immune response elicited by the vaccine does not appear to compromise vaccination efficacy in terms of reduction in the number of viraemic animals [28]. Noteworthy, extremely high MDA levels can even interfere the vaccine efficacy in terms of improvement of ADWG [27]. Nevertheless, vaccine immune response cannot be solely measured by seroconversion, as both humoral and cell-mediated responses contribute to protection against PCV2 [6,25].

The objective of this study was to assess the effect of high and low PCV2 MDAs on the efficacy against PCV2 of a new ready-to-use combined vaccine based on PCV2d genotype and *M. hyopneumoniae* in piglets experimentally inoculated with PCV2b.

## 2. Materials and Methods

### 2.1. Animal Selection and Housing

A total of 150 2-week-old piglets (Large White-Landrace and Duroc crossbreed coming from non-vaccinated sows) were screened with the objective of selecting enough animals with low and high PCV2 antibody levels. Piglets came from a farm negative for porcine reproductive and respiratory syndrome virus (PRRSV) and seropositive against *M. hyopneumoniae*, *Glaesserella parasuis* and *Actinobacillus pleuropneumoniae*. Forty-eight clinically healthy piglets were taken to complete the experimental study. The selected piglets were negative to PRRSV and PCV2 by RT-qPCR or qPCR, respectively, and were distributed according to their PCV2 ELISA S/P values (Ingezim Circo IgG 11.PCV.K1^®^, Gold Standard Diagnostics, Madrid, Spain) into two experimental groups of 24 animals. Group L comprised pigs with the lowest ELISA S/P ratios (ranging between 0.189 and 0.435), while group H included those with the highest S/P ratios (ranging between 1.073 and 1.511). Afterwards, groups L and H were further divided into non-vaccinated (H-NV and L-NV) and vaccinated (H-V and L-V) subgroups (*n* = 12 each). These piglets were transported to A.M. Animalia Bianya S.L. experimental facilities (Girona, Spain) at two weeks of age. Housing conditions, feeding system, feed characteristics and health management were the same for all groups. This study was approved by the Ethics Commission of Generalitat de Catalunya (Spain) through A.M. Animalia Bianya S.L., under the reference 028/23.

### 2.2. Experimental Study Design and Sample Collection

**Figure 1:**
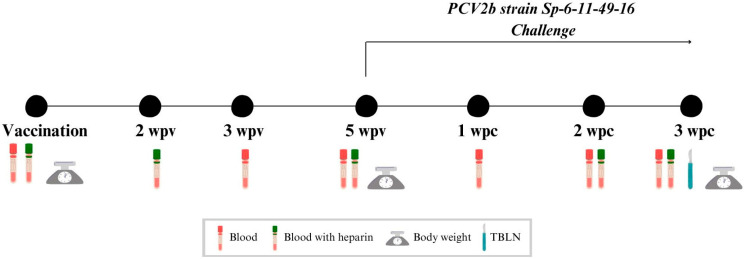
Scheme of the study design. TBLN: tracheobronchial lymph node; wpv: weeks post-vaccination; wpc: weeks post-challenge.

Experimental design is shown in Figure 1. After a one-week acclimatation period, at three weeks of age, H-V and L-V piglets received intramuscularly one dose of 2 mL of Cirbloc^®^ M Hyo (Ceva Santé Animale, Libourne, France) (batch number 002NG1N) on the right side of the neck muscles following the manufacturer’s recommendations, while H-NV and L-NV were injected with 2 mL of phosphate buffered saline (PBS) in an equivalent manner. Five weeks post-vaccination or PBS injection (5 wpv), all animals were challenged intranasally with 3 mL of inoculum (1.5 mL in each nostril) containing 10^4,73^ TCID_50_/mL of PCV2b strain Sp-6-11-49-16 (Genbank accession number: EF647673.1). This study concluded with the euthanasia and necropsy of all animals three weeks post-challenge (3 wpc).

Blood samples were collected at vaccination, at 3 and 5 wpv and at 1, 2 and 3 wpc to obtain serum (Figure 1). Heparinised blood samples were collected from half of the individuals of each group (*n* = 24; 6 per subgroup) at vaccination, at 2 and 5 wpv and at 2 and 3 wpc to obtain peripheral blood mononuclear cells (PBMCs). A tracheobronchial lymph node (TBLN) was collected from each animal at necropsy (3 wpc), both fresh (to perform PCV2 qPCR) and fixed by immersion in 10% buffered formalin (for histopathology and in situ hybridisation (ISH) analyses).

Additionally, all animals were weighted at vaccination at 5 wpv and 3 wpc. Average daily weight gain (ADWG) was calculated based on the body weight (BW) at three different periods: from vaccination to challenge, from challenge to necropsy and from vaccination to necropsy.

### 2.3. DNA Extraction and Detection of PCV2 by qPCR

DNA extraction and PCV2 detection by qPCR were performed on serum samples and supernatant of macerated TBLN individually. DNA was extracted from 200 µL of serum or tissue supernatant using the MagMAX^®^ Pathogen RNA/DNA Kit (Applied Biosystems, CA, USA) according to the manufacturer′s guidelines. Each extraction process and plate included negative controls to check for potential contamination.

To detect and quantify PCV2, the LSI VetMAX^TM^ Porcine Circovirus Type 2 Quantification qPCR assay (Thermo Fisher Scientific, Waltham, MA, USA) was used. Every qPCR plate contained a standard curve, negative controls and an internal positive control (IPC) to ensure the reliability of extraction and amplification processes. The limit of quantification (LOQ) was set at 1.0 × 10^4^ genome copies/mL, and the limit of detection (LOD) was set at 4 × 10^3^ for both serum and tissue supernatant, as previously described [29]. qPCR results were then log_10_ transformed and classified in three categories: below LOD (which included from undetermined to <3.6 log_10_ PCV2 DNA copies/mL values), positive but not quantifiable results (between LOD and LOQ, namely from 3.6 to 4.0 log_10_ PCV2 DNA copies/mL) and positive and quantifiable results (>4.0 log_10_ PCV2 DNA copies/mL). For statistical purposes, the following assumptions were made based on a previous study [29]: the undetermined and LOD results were assigned a value equivalent to half of the LOD (which corresponds to 3.3 log_10_), and positive but not quantifiable results were assigned the LOQ value (4.0 log_10_). Additionally, the area under the curve (AUC) of viral load was calculated for each animal from 5 wpv to 3 wpc.

### 2.4. PCV2 Antibody Levels Measured by PCV2 IgG ELISA

PCV2 antibody levels were determined in serum samples using a commercial indirect ELISA kit (Ingezim Circo IgG 11.PCV.K1^®^, Gold Standard Diagnostics, Madrid, Spain) according to manufacturer’s instructions. From each animal, serum samples taken at different time points were tested by duplicate on the same ELISA plate. Optical density (OD) readings were taken at 450 nm using the Sunrise™ reader (Tecan, Männendorf, Switzerland). Results were reported as the mean S/P ratio (sample OD/positive control OD) per tested serum.

### 2.5. Histopathology and In Situ Hybridisation

Formalin-fixed TBLNs were dehydrated and embedded in paraffin. From each paraffin block, 4 µm thick sections were cut, stained with haematoxylin-eosin (HE) and examined for the presence of lesions indicative of PCV2 infection, such as lymphocyte depletion (LD) and histiocytic replacement (HR). Additionally, a contiguous section was prepared to detect the PCV2 genome by ISH using RNAscope^®^ Technology (ACDBio, Newark, CA, USA) according to the manufacturer’s procedures and as previously described [30,31]. Afterwards, LD, HR and the amount of PCV2 genome were scored from 0 (no lesions or no staining) to 3 (severe lesions or widespread antigen distribution) [30].

### 2.6. Peripheral Blood Mononuclear Cells (PBMCs) Isolation and Stimulation

PBMCs were extracted from blood samples collected in heparinized tubes using density gradient centrifugation with Histopaque^®^ 1.077 (Sigma, Madrid, Spain). The isolated PBMCs were washed and resuspended in complete RPMI 1640 medium (Corning, Corning, NY, USA) (cRPMI) supplemented with 10% foetal bovine serum (FBS) (Sigma, Madrid, Spain). Cell viability was determined using Trypan blue staining. PBMCs were then plated in 96-well plates at a density of 1 × 10^6^ cells per well and incubated with baculovirus-expressed PCV2 Cap protein (0.6 μg/mL final concentration per well, kindly provided by Gold Standard Diagnostics, Madrid, Spain), phytohemagglutinin (positive control, 10 μg/mL final concentration per well, Sigma, Madrid, Spain) or cRPMI with 10% FBS (negative control) for 24 h at 37 °C in a humidified 5% CO_2_ atmosphere. After incubation, the plates were centrifuged, and the cell culture supernatants were collected and stored at −80 °C for further analysis [32,33,34].

### 2.7. Multiplex Immunoassay for the Quantification of Cytokines

Supernatant samples from PBMCs were examined using the ProcartaPlex™ Porcine Cytokine & Chemokine Panel 1 (Invitrogen, Thermo Fisher Scientific, Newark, MA, USA), following manufacturer’s guidelines. This multiplex immunoassay uses Luminex^®^ xMAP technology to measure nine cytokines: IFN-α, IFN-γ, IL-12, TNF-α, IL-1β, IL-8, IL-4, IL-6, and IL-10. The cytokine levels were quantified with a MAGPIX^®^ analyser (Luminex Corporation, Austin, TX, USA) and interpreted using xPONENT^®^ 4.2 software (Luminex Corporation, Austin, TX, USA), based on standard curves. For calculating PCV2-specific cytokine secretion (pg/mL), the cytokine concentrations in supernatants from PBMCs cultured with cRPMI (background) were subtracted from those in supernatants from PBMCs stimulated with the PCV2 Cap protein [33].

### 2.8. Statistical Analyses

The normal distribution of all studied quantitative variables (BW, ADWG, PCV2 ELISA S/P ratios, PCV2 log_10_ load in serum and tissue supernatant, AUC and cytokine amounts) was verified by the Shapiro–Wilk test. The different parameters were first analysed between V and NV to assess vaccination efficacy and subsequently among H-V, H-NV, L-V and L-NV to study the effect of MDA on this efficacy.

For the V and NV analyses, BW and ADWG differences for each time point were analysed using a *t*-test, and AUC differences were assessed using a Mann–Whitney test. The percentage of PCV2 qPCR-positive serum samples (quantifiable and non-quantifiable results) at 1, 2 and 3 wpc, as well as the PCV2 qPCR-positive TBLN samples, were compared using the Fisher test. PCV2 ELISA S/P ratio and PCV2 log_10_ loads between groups and time points were analysed using a two-way ANOVA test and Tukey’s multiple comparison test. Additionally, the effect of MDA levels on the seroconversion of piglets was assessed by calculating the Pearson correlation coefficient was calculated between PCV2 S/P ratios at vaccination and the Delta value across the different periods: from vaccination to 3 wpv, from vaccination to 5 wpv and from 3 wpv to 5 wpv. Finally, the comparison of each cytokine concentration was performed between groups and time points using a mixed effects model and Tukey’s multiple comparison test.

For all group comparisons (H-V, H-NV, L-V and L-NV), BW and ADWG differences for each time point were analysed using the one-way ANOVA test and Tukey’s multiple comparison test, whereas AUC differences were assessed using a Kruskal–Wallis test and a Dunn’s multiple comparisons test. The rest of the parameters were analysed as described for the V and NV groups.

Statistical analyses and graphics were performed using Graphpad^®^ (Graphpad Software, Boston, MA, USA). The significance level (*p*-value) was set at 0.05, and the trend towards statistical significance was set as 0.1.

## 3. Results

The results (except those from the clinical assessment, which are presented together) were first analysed by comparing vaccinated (V) and non-vaccinated (NV) groups and subsequently among the high MDA vaccinated (H-V), high MDA non-vaccinated (H-NV), low MDA vaccinated (L-V) and low MDA non-vaccinated (L-NV) groups.

### 3.1. Clinical Assessment

During this study, four animals (three from H-NV group and one from L-V group) died due to causes unrelated to the vaccination or challenge procedures. One was due to an intestinal intussusception, another due to a haemorrhagic enteritis by *Escherichia coli*, and the other two died with signs and gross lesions compatible with septicaemia (cyanosis and petechial haemorrhages). The rest of the animals remained healthy during the whole experimental period, without evident clinical signs after PCV2 challenge.

### 3.2. Comparison Between V and NV Groups

#### 3.2.1. BW and ADWG

The differences between the V and NV groups on BW and ADWG were not statistically significant at any time point of this study (Table 1).

#### 3.2.2. Dynamics of PCV2 IgG Antibody Levels

**Figure 2:**
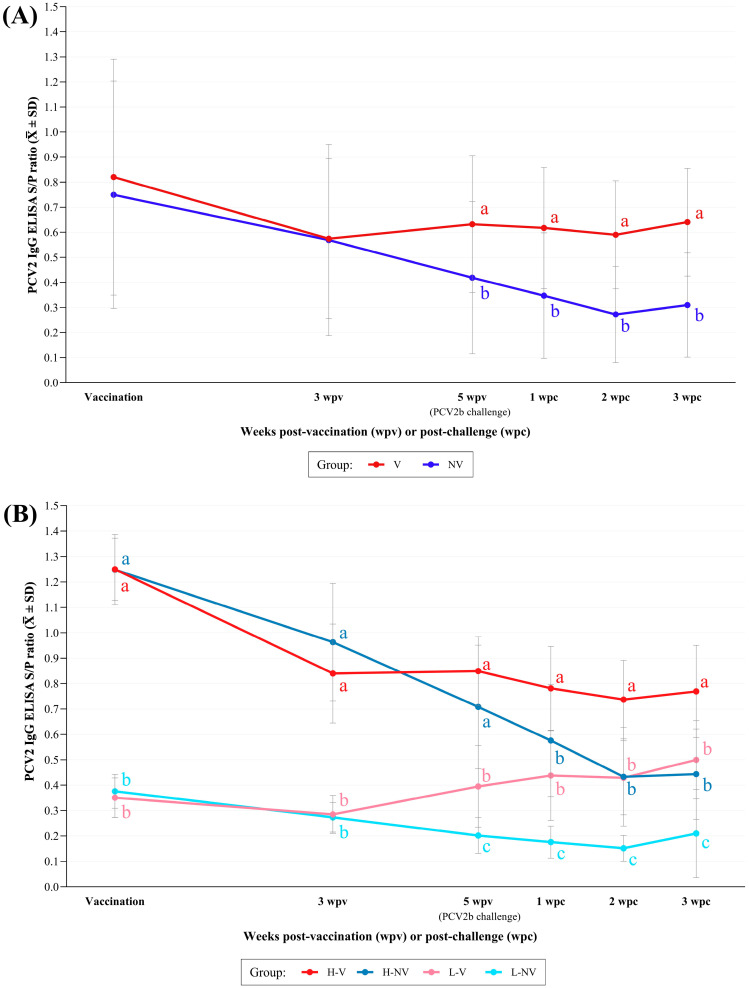
Mean (± SD) PCV2 IgG ELISA S/P ratio obtained at different time points between the V vs. NV (**A**) and the H-V, H-NV, L-V and L-NV (**B**) groups. Different letters in superscript indicate statistically significant differences between groups at each time point (*p* < 0.05). H: high; L: low; V: vaccinated; NV: not vaccinated; wpv: weeks post-vaccination; wpc: weeks post-challenge.

**Figure A1:**
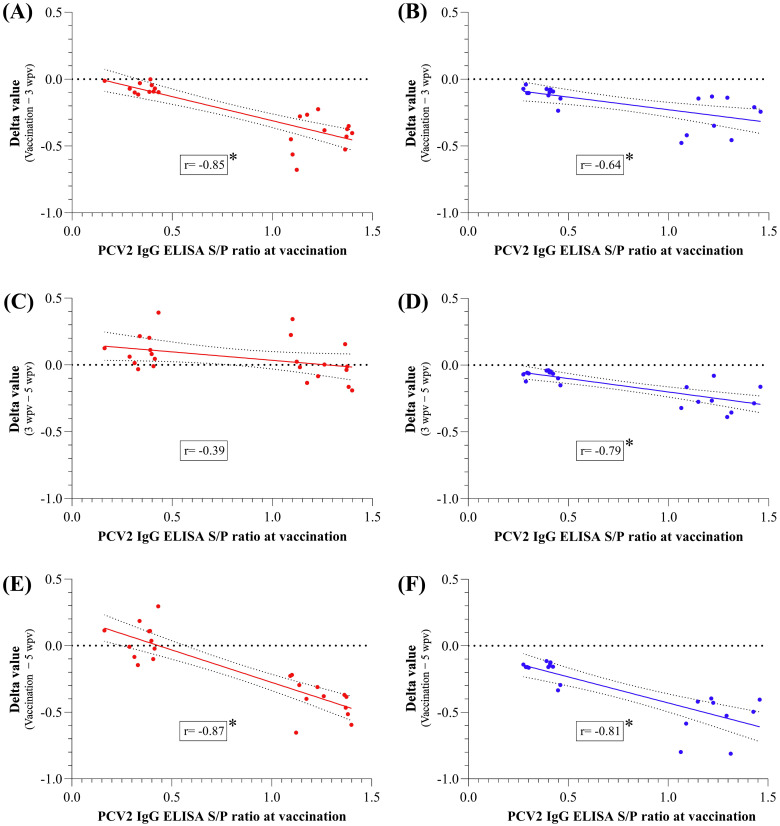
Pearson correlation coefficients between PCV2 antibody levels at vaccination and the Delta value from vaccination to 3 wpv (**A**,**B**), 3 to 5 wpv (**C**,**D**) and vaccination to 5 wpv (challenge) (**E**,**F**) for V animals, shown in red (**A**,**C**,**E**), and NV animals, shown in blue (**B**,**D**,**F**). * indicates a statistically significant correlation (*p* < 0.05). wpv: weeks post-vaccination; NV: not vaccinated; V: vaccinated.

The vaccinated animals showed significantly higher PCV2 S/P ELISA values from 5 wpv to necropsy than the NV ones. In fact, this latter group showed a statistically significant decline (*p* < 0.05) in antibody levels over the time until 2wpc (Figure 2A). Significant (*p* < 0.05) negative correlations were found between PCV2 ELISA S/P ratios at vaccination and the corresponding Delta values from vaccination to 3 wpv, and from vaccination to 5 wpv for both groups; from 3 to 5 wpv it was only significant for NV group (Figure A1). After 5 wpv, the V group exhibited relatively stable antibody levels.

#### 3.2.3. PCV2 Infection Dynamics

**Figure 3:**
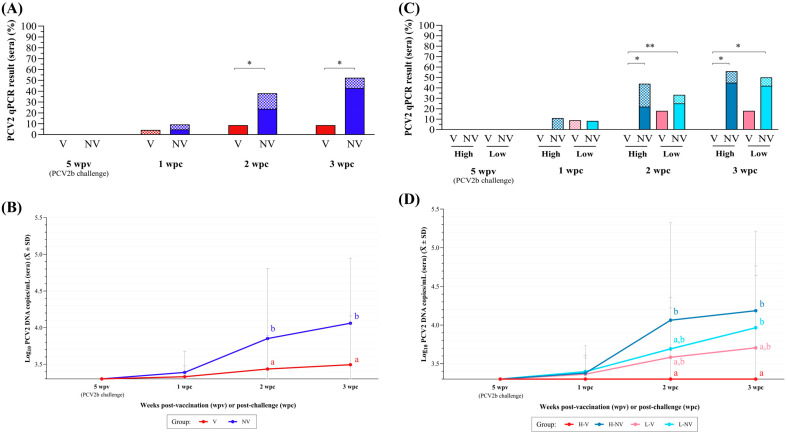
PCV2 qPCR results obtained in sera weekly after PCV2b challenge. (**A**) Percentage of positive samples in animals from V (in red) and NV (in blue) groups, shown as quantifiable (solid-coloured portion) and non-quantifiable or below the LOQ (checkered portion). (**B**) Mean (± SD) log_10_ PCV2 DNA copies/mL of serum for V (in red) and NV (in blue) groups. (**C**) Percentage of positive samples in animals from H-V (red), H-NV (dark blue), L-V (pink) and L-NV (light blue), shown as quantifiable (solid-coloured portion) and non-quantifiable or below the LOQ (checkered portion). (**D**) Mean (± SD) log_10_ PCV2 DNA copies/mL of serum for H-V (red), H-NV (dark blue), L-V (pink) and L-NV (light blue) groups. In subfigures (**A**,**C**), * indicates statistically significant differences between groups at each time point (*p* < 0.05), and ** indicate a trend towards statistical significance (*p* < 0.1). In subfigures (**B**,**D**), different superscript letters indicate statistically significant differences between groups in mean log_10_ PCV2 copies/mL at each time point (*p* < 0.05). PCV2: porcine circovirus 2; wpv: weeks post-vaccination; wpc: weeks post-challenge; H: high; L: low; NV: not vaccinated; V: vaccinated.

**Figure 4:**
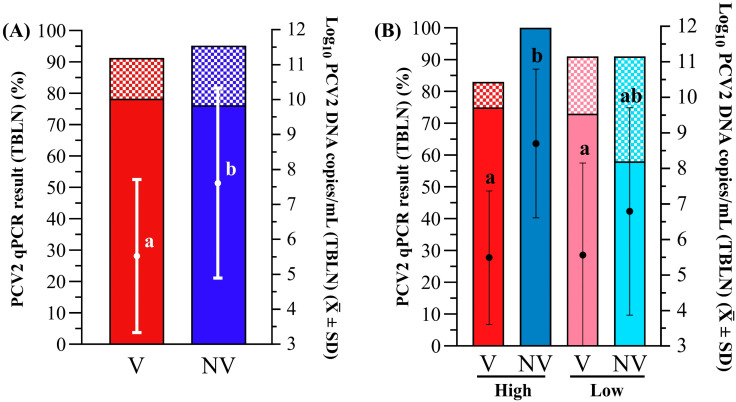
PCV2 qPCR results obtained in TBLN for (**A**) V (in red) vs. NV (in blue) and (**B**) H-V (red), H-NV (dark blue), L-V (pink) and L-NV (light blue) groups. Left Y axes indicate the percentage (in bars) of positive quantifiable (solid-coloured portion) and non-quantifiable (checkered portion) samples. Right Y axes refer to the mean log_10_ PCV2 DNA copies/mL of tissue supernatant (± SD), shown as individual dots. Different superscript letters indicate statistically significant differences in mean log_10_ PCV2 DNA copies/mL of tissue supernatant between groups (*p* < 0.05). PCV2: porcine circovirus 2; TBLN: tracheobronchial lymph node; H: high; L: low; V: vaccinated; NV: not vaccinated.

Animals from both groups remained PCV2 qPCR negative during the period from vaccination to challenge. The V group showed a significantly lower percentage of PCV2 qPCR-positive animals (Figure 3A) and lower mean viral load (Figure 3B) in serum at 2 and 3 wpc when compared to the NV group (*p* < 0.05). These differences were coupled with a significantly lower AUC after challenge in the V group (10.2 ± 0.8) compared to the NV group (10.9 ± 1.5) (*p* < 0.05). The PCV2 load in TBLN was also significantly higher in the NV group compared to the V group (*p* < 0.05), but all groups had a similar (*p* > 0.05) percentage of qPCR PCV2 positivity of TBLN between groups (Figure 4A).

#### 3.2.4. Lesion Assessment and PCV2 Antigen Detection in TBLN

Three animals out of 21 from the NV group had lesions of HR (scores 1 or 2). The one with moderate HR also had an LD score of 1.

**Figure 5:**
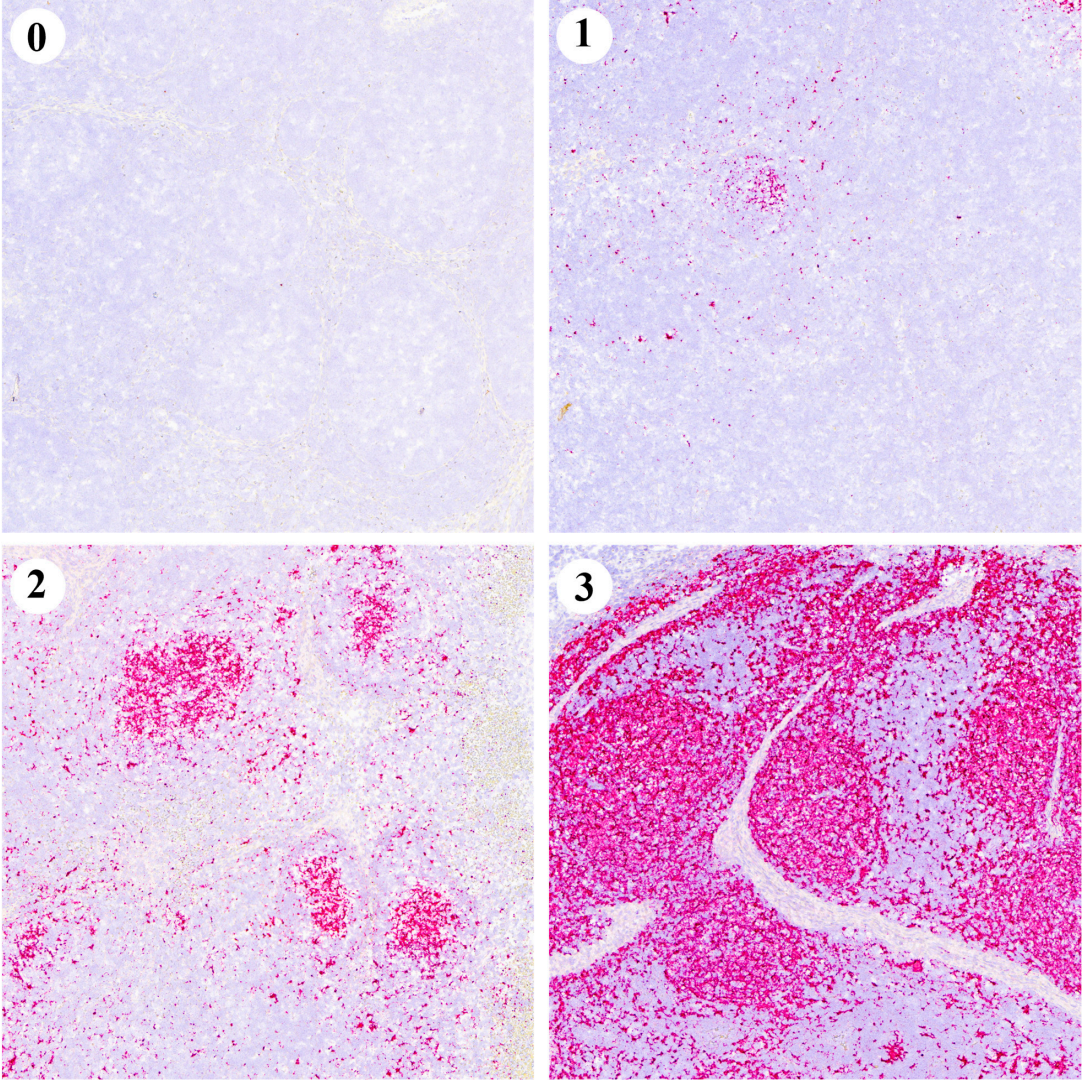
Representative images of the PCV2 in situ hybridisation (ISH) scoring system (0–3) from TBLNs. Each red dot represents a hybridisation signal indicating the presence of the PCV2 genome.

Examples of the ISH scoring values are depicted in Figure 5. Globally, the V group had a statistically higher percentage of animals scored 0 (73.9%), compared to the NV group (33.3%) (*p* < 0.05). Conversely, the NV group had a statistically higher percentage of animals scored 3 (19.0%) compared to the V group (0.0%) (*p* < 0.05) (Table 2).

#### 3.2.5. Cytokine Concentration

**Figure 6:**
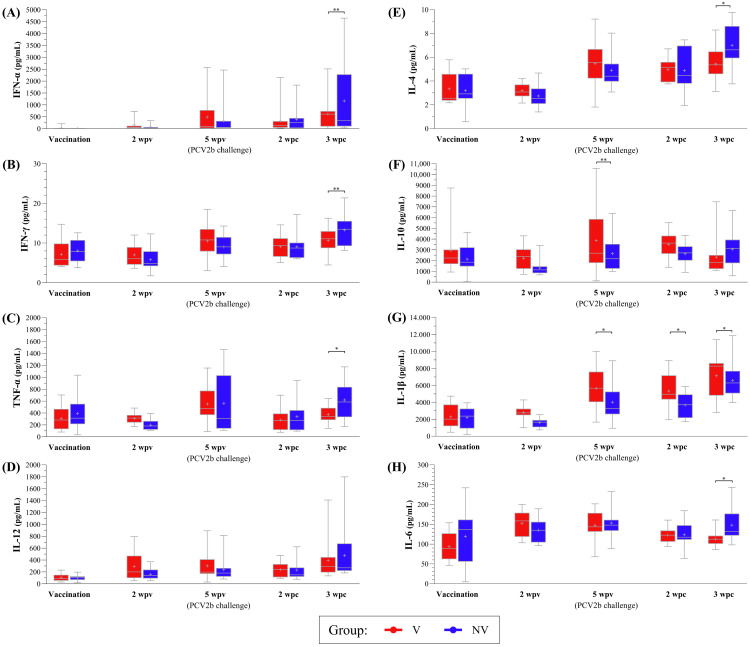
Boxplots showing the median (horizontal line) and mean (cross) values of PCV2-specific cytokine secretion (IFN-α, IFN-γ, TNF-α, IL-12, IL-4, IL-10, IL-1β and IL-6, labelled from (**A**–**H**), respectively) (pg/mL) in PBMC supernatant samples from V and NV groups at different time points. * indicates statistically significant differences between groups for each time point (*p* < 0.05). ** indicate a trend towards statistical significance (*p* < 0.1). IFN: interferon; IL: interleukin; TNF: tumour necrosis factor; PCV2: porcine circovirus 2; NV: not vaccinated; V: vaccinated.

The vaccinated animals exhibited an increasing trend from vaccination to 5 wpv for IFN-α and IFN-γ (*p* < 0.05), as well as for TNF-α and IL-12 (*p* < 0.1), reaching stability from 5 wpv onwards. However, for the NV group, a significant increasing trend relative to placebo administration was observed at 3 wpc for the three cytokines (*p* < 0.05), except for TNF-α, which showed significance when comparing 2 wpv with 3 wpc (*p* < 0.05). The values of the NV piglets were higher across the mentioned cytokines at 3 wpc compared to those of the V animals, showing significance for TNF-α (*p* < 0.05) and a trend for IFN-α and IFN-γ (*p* < 0.1) (Figure 6A–D).

IL-4 levels followed a similar pattern as seen with the previous cytokines for the V (increasing significantly from vaccination to 5 wpv, *p* < 0.05) and NV piglets (with an increasing trend from vaccination to 3 wpc, *p* < 0.05, which was also higher than that of the V group at that time, *p* < 0.05) (Figure 6E). However, IL-10 levels remained mostly stable in both V and NV groups from vaccination to 3 wpc, with a higher concentration observed in the V group, compared to the NV group, only at 5 wpv (*p* < 0.1) (Figure 6F).

Regarding the remaining cytokines, both V and NV animals showed significant increases in IL-1β from vaccination to 5 wpv and 3 wpc, with the V pigs displaying higher concentrations at 5 wpv and 2–3 wpc (*p* < 0.05) (Figure 6G). IL-6 levels increased significantly in V animals at 5 wpv (*p* < 0.05) before declining, whereas the NV pigs showed a trend towards an increase by 5 wpv, with levels becoming significantly higher than those of the V animals at 3 wpc (*p* < 0.05) (Figure 6H).

Finally, IL-8 results could not be interpreted as these values were out of range for most of the samples.

### 3.3. Comparison Among H-V, H-NV, L-V and L-NV Groups

#### 3.3.1. BW and ADWG

Differences in BW were not statistically significant between groups (Table 1). The animals from the H-NV group showed significantly higher ADWG from challenge to necropsy when compared to their L-NV counterparts (*p* < 0.05) (Table 1).

#### 3.3.2. Dynamics of PCV2 IgG Antibody Levels 

The H-V animals showed significantly higher values than the other three groups from 1 wpc onwards (Figure 2B). In fact, the L-NV group exhibited the lowest S/P ELISA values, being statistically significant compared to the other three groups from 5 wpv until necropsy (*p* < 0.05). There were no statistically significant correlations between PCV2 ELISA S/P ratios at vaccination and the corresponding Delta values from vaccination to 3 wpv, from vaccination to 5 wpv or from 3 to 5 wpv for any of the groups. However, a trend towards significance (*p* < 0.1) was observed from vaccination to 3 wpv in the L-NV group (r = −0.53) and from vaccination to 5 wpv in the H-V group (r = −0.53).

#### 3.3.3. PCV2 Infection Dynamics

**Figure A2:**
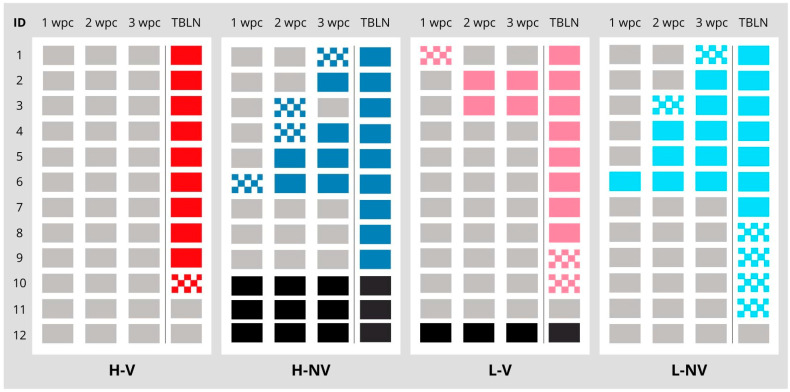
Distribution of PCV2 qPCR results for individual animals in each experimental group (H-V in red, H-NV in dark blue, L-V in pink and L-NV in light blue) from 1 to 3 wpc, as well as in serum and in tissue supernatant from TBLNs. Each row represents an individual animal’s qPCR result, with columns indicating time points and TBLN results. The results are interpreted as follows: positive quantifiable (solid coloured), below the LOQ (checkered) and below the LOD (white). Black boxes indicate missing data of animals that died during this study. H: high; L: low; V: vaccinated; NV: not vaccinated; POS: positive; LOQ: limit of quantification; LOD: limit of detection; TBLN: tracheobronchial lymph node; wpc: weeks post-challenge.

None of the animals from the H-V group had a viral load higher than the LOD throughout this study. In contrast, the H-NV group showed a significantly higher percentage of viraemic piglets when compared to the H-V group at 2 and 3 wpc (*p* < 0.05) (Figure 3C). At necropsy (3 wpc), the L-NV group also had a significantly higher percentage of viraemic piglets when compared to the H-V group (*p* < 0.05). The same statistically significant differences were observed when the viral load was compared (Figure 3D). In terms of AUC, the H-V pigs had statistically lower AUC (9.9 ± 0.0), as the undetermined values were assigned half of the LOD value, compared to the H-NV (11.2 ± 1.8) (*p* < 0.05) and L-NV (10.7 ± 1.2) (*p* < 0.1) animals, with no significant differences observed when compared to L-V pigs (10.5 ± 1.1). Notably, despite the lack of differences in positivity or viral load between the L-V and L-NV groups, the number of pigs ever viraemic (at least positive at one time point) was lower in the L-V group (3/11, 27.3% compared to the L-NV group (6/12, 50%) (Figure A2).

The mean PCV2 load in the TBLNs was also significantly higher in the H-NV group compared to both the H-V and L-V groups (*p* < 0.05), with no statistically significant differences in percentages of PCV2-positive TBLN samples observed among groups (Figure 4B).

#### 3.3.4. Lesion Assessment and PCV2 Antigen Detection in TBLN Samples

Two out of 12 animals from the L-NV group (with a score of 1) and one out of 9 from the H-NV group (with a score of 2) exhibited HR. The latter also exhibited LD with a score of 1.

Regarding ISH, both vaccinated groups had a higher percentage of animals scored 0 (H-V with 66.7% and L-V with 81.8%) (*p* < 0.05) compared with H-NV (11.1%). The H-NV group also had 33.3% of animals with a score of 3, showing a trend towards statistical significance (*p* < 0.1) compared to the V groups (Table 2).

#### 3.3.5. Cytokine Concentration

**Figure 7:**
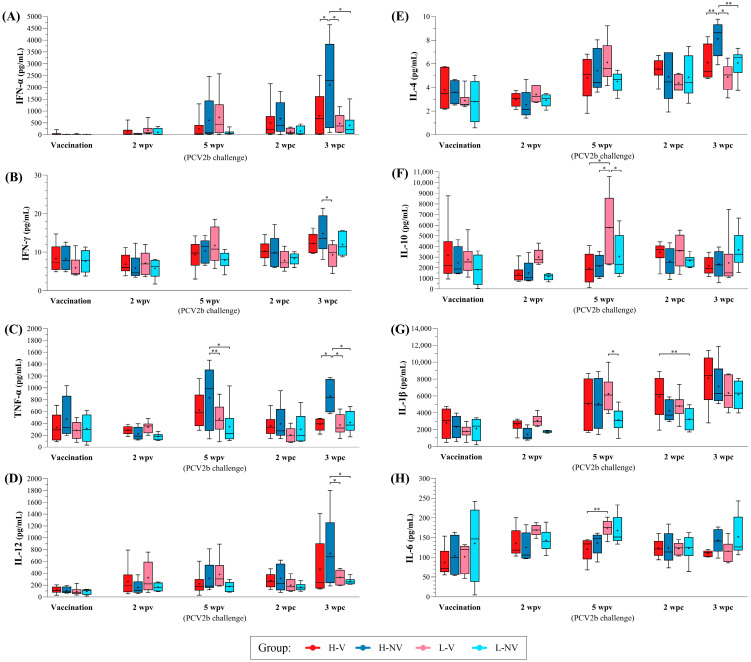
Boxplots showing the median (horizontal line) and mean (cross) values of PCV2-specific cytokine secretion (IFN-α, IFN-γ, TNF-α, IL-12, IL-4, IL-10, IL-1β and IL-6, labelled from (**A**–**H**), respectively) (pg/mL) in PBMC supernatant samples from H-V, H-NV, L-V and L-NV groups at different time points. * indicates statistically significant differences between groups for each time point (*p* < 0.05). ** indicate a trend towards statistical significance (*p* < 0.1). IFN: interferon; IL: interleukin; TNF: tumour necrosis factor; PCV2: porcine circovirus 2; H: high; L: low; NV: not vaccinated; V: vaccinated.

The H-NV group exhibited higher levels of IFN-α, IFN-γ, TNF-α and IL-12 at 3 wpc compared to the other groups. TNF-α levels were also significantly higher at 5 wpv in this group, both compared to the other groups (*p* < 0.05) and relative to levels at vaccination (*p* < 0.05) (Figure 7A–D). In contrast, the L-NV group remained mostly stable for the mentioned cytokines, except for IFN-γ, which showed a significant increase from vaccination to 3 wpc (*p* < 0.05). Regarding the L-V group, a significant increase in IFN-γ and IL-12 (*p* < 0.05) was observed from vaccination to 5 wpv, while in the in the H-V group, both cytokines increased significantly from vaccination to 3 wpc (*p* < 0.05).

Regarding IL-4 and IL-10, the L-V group showed a significant increase in both from vaccination to 5 wpv (*p* < 0.05) (Figure 7E,F). Similarly, the H-NV pigs exhibited a significant rise in IL-4 from 5 wpv to 3 wpc (*p* < 0.05), although all groups also showed significant increases from vaccination to challenge (*p* < 0.05). In contrast, the level of IL-10 remained stable throughout this study in the H-V, H-NV and L-NV groups.

Finally, all groups, except the L-NV ones, showed significant increases in IL-1β from vaccination to 5 wpv and to 3 wpc (*p* < 0.05) (Figure 7G). Regarding IL-6, both H-V and L-V showed significant increases from vaccination to 2 wpv (*p* < 0.05). In the L-V group, this increase was followed by a significant decrease at 3 wpc (*p* < 0.05), while in the H-NV and L-NV groups the IL-6 levels remained mostly stable (Figure 7H).

## 4. Discussion

The current study aimed to evaluate how different levels of MDA (high and low) against PCV2 influence the efficacy of a new, ready-to-use combined vaccine containing the PCV2d genotype and *M. hyopneumoniae* in piglets that were vaccinated at 3 weeks of age and experimentally infected 5 wpv with a PCV2b strain. Specifically, this work was designed to study such efficacy based on a number of parameters, including ADWG, virology, pathology and humoral and cellular immune responses in a subclinical PCV2 infection scenario.

When assessing the humoral immune response, the V group maintained significantly higher S/P ratio levels than the NV group from 5 wpv to 3 wpc, results that were also consistent with the significantly lower PCV2 load in serum and in the TBLN samples, a reduced AUC, as well as a lower percentage of qPCR-positive animals in serum and the absence of animals scored 3 by ISH. These results align both with the efficacy observed in previous studies using other PCV2 combined ready-to-mix and/or ready-to-use vaccines [29,35] and with the results of a recent study evaluating the same vaccine [36]. The lack of differences in BW or ADWG among the V and NV groups is probably not surprising, taking into account that the number of animals tested under experimental conditions was limited, the infection outcome was subclinical, and the time evaluated (3 weeks from challenge to necropsy) was short [37,38]. Notably, numerical differences in ADWG were observed among groups, and these differences were significant between the H-NV and the L-NV pigs. Such result reinforces the significant contribution of maternally derived immunity to the protective efficacy against PCV2 infection [39]. However, the pathological and virological results among these two groups were not significantly different.

The present study demonstrated genotype cross-protection effects, since the challenge was conducted using PCV2b, and the tested vaccine contained PCV2d. This result is coherent with findings for other PCV2 vaccines with varying formulations, as summarized in a recent review [20], and is further supported by a newly published study performed using the same vaccine [40].

In the present study, PCV2 infection was monitored longitudinally in serum by qPCR, with viral loads below LOQ at 1 to 3 wpc found in several pigs. One possible explanation for this limited detection rate could be the lower viral dose used for inoculation (3 mL of 10^4,73^ TCID_50_/mL) compared to previous studies in which a similar PCV2b isolates were used [41,42]. Another potential factor that influences the outcome of PCV2 infection is host genetics, i.e., Landrace pigs were found to be more susceptible than Large White or Duroc pigs [43,44,45]. Supporting this hypothesis, another study [46] also found genetic differences to be significant in determining susceptibility to PCV2 infection among pig breeds, further demonstrating this relationship [7]. In contrast, the TBLN samples tested positive for PCV2 by qPCR in almost all animals. The selection of the TBLN for viral load assessment stems from its role as a lymphoid tissue involved in PCV2 pathogenesis [4,47]. The TBLN has been reported to exhibit higher viral loads than other lymph nodes under experimental conditions [48]. Moreover, previous studies have shown a 2–3 log_10_ difference between viral loads in blood and lymph nodes [4,7]. In fact, in the present study, the NV groups showed significantly higher viral loads in the TBLN compared to the V animals, with some reaching 10–11 Log_10_ PCV2 DNA copies/mL.

Additionally, none of the animals displayed clinical signs compatible with PCV2-systemic disease, a fact that correlates with serum viral loads below the classical threshold of 10^7^ PCV2 copies/mL described in several works [49,50] and with the limited number of animals with histopathological lesions (only three animals and with mild to moderate lesions). On the contrary, a high percentage of infected animals from NV groups showed abundant PCV2 antigen (ISH score 3) in the TBLN samples by RNAscope^®^ technology. This discrepancy reflects the high sensitivity of this latter technique when compared with other techniques, such as IHC or the traditional ISH [30]. These less sensitive techniques are typically able to confirm infection and disease in animals with moderate to high viral loads [47,51].

MDAs are known to provide piglet protection against PCV2 infection early in life; however, high or very high levels of antibodies at the moment of vaccination may interfere with the humoral immune response elicited by the vaccine and the ADWG [27]. Notably, declining PCV2 antibody level trends were observed across all groups from vaccination to 3 wpv, likely due to the natural waning of MDAs. The phenomenon has been shown to occur between 4 and 12 weeks of age and has been reported in several studies examining PCV2 vaccination [37,52,53,54].

The L-V group showed seroconversion from 3 to 5 wpv, a response that was less pronounced in the H-V group, as expected, as high MDA levels jeopardize the vaccine-induced seroconversion in piglets [28]. However, a mild seroconversion after vaccination in the presence of MDAs should not be considered as a negative indicator for the vaccination effectiveness [55,56].

In the present study, the H-V group exhibited the lowest viral loads, with all animals remaining below the limit of quantification (LOQ) across all time points in both sera and TBLN. This group also had a reduced viral AUC and no ISH score of 3 in TBLN, showing no significant differences compared to the L-V group. In contrast, the H-NV group showed the highest viral loads and AUC values, together with higher ISH scores (6 out of 9 animals had scores of 2 and 3), followed by the L-NV group. These findings highlight the importance of vaccination in reducing PCV2 viral replication [4,7] and indicate that vaccination efficacy was not jeopardized despite high values of MDA, as also seen in previous studies [28,54,57,58]. In this regard, the reduction in viraemia and tissue viral load observed in vaccinated animals supports the concept of virological protection, a term commonly used in vaccine efficacy studies to describe partial protection based on virological outcomes rather than on the complete prevention of infection [59,60,61,62].

Both cellular and humoral responses are considered of great importance for the PCV2 control, as PCV2 antibodies are not capable to confer full protection against this viral infection [63,64,65]. Indeed, the level of neutralizing antibodies has been correlated with viral replication, lesion severity and disease development in PCV2-infected pigs [63,66]. In addition, MDAs have been shown to protect piglets by reducing the probability of viraemia [39,67,68]. Regarding cellular immunity, T helper (Th) 1 cytokines promote the ability to fight intracellular pathogens such as PCV2, and Th2 cytokines play a role in neutralizing viruses, but are more closely associated with B-cell proliferation and specific antibody formation [69,70,71]. Here, the cellular responses were studied using a multiplex immunoassay, which allowed simultaneous quantification of cytokines in PBMCs and allowed the assessment of IFN-α, IFN-γ, TNF-α and IL-12, IL-4, IL-10, IL-1β and IL-6.

In terms of cytokine response, the NV group (especially H-NV) exhibited increases in IFN-γ, IFN-α and TNF-α post-challenge, probably suggesting an inflammatory response in the absence of previous vaccination. In contrast, the V animals displayed relatively stable or moderate increase in these cytokines after the PCV2b challenge, likely due to lower levels of viral replication. Such a response aligns with previous findings, in which vaccination triggered an early, strong IFN-γ-secreting cell response, while non-vaccinated animals exhibited higher IFN-γ levels later [72]. These results may indicate that vaccination fosters long-lasting immunity through memory T cells and IFN-γ-secreting cells, potentially preventing the onset of infection. The observed increase in IL-12 could further support IFN-γ production in NV animals by 3 wpc, reflecting a heightened immune activation following infection [73,74]. This amplified response of IL-12 was predominantly seen in the H-NV animals, which also showed concurrent elevation of TNF-α alongside IFN-γ. Moreover, PCV2 infection in vivo has been shown to induce IFN-α secretion, establishing an antiviral state that may enhance the defence mechanism [71]. In our study, the NV group showed higher amounts of IFN-α by 3 wpc, probably promoted by higher viral replication compared to the V group.

Following a similar pattern, the V pigs showed a significant increase in IL-4 by 5 wpv. This cytokine has been reported to be upregulated in PBMCs from vaccinated pigs [75], potentially indicating that vaccination primed an effective humoral response. In contrast, the NV group exhibited a rise in IL-4 only by 3 wpc. This response, especially evident in the H-NV pigs, may reflect a compensatory immune mechanism aimed at countering viral load post-infection, as this group exhibited the highest viral loads. On the other hand, IL-10 production was notably higher in the L-V group at 5 wpv. This cytokine is often linked to immune dysregulation in animals with PCV2-systemic disease [71,76].

Although IL-1β had significant higher concentrations in the V group from 5 wpv onwards, when split for the subgroups, the differences were not conclusive. Regarding IL-6, a well-known pro-inflammatory cytokine, it was significantly increased in the NV animals at 3 wpc. This finding is in line with the higher viral load observed in this group and agrees with previous studies reporting that PCV2 infection can modulate IL-6 expression, with increased levels linked to enhanced inflammatory responses depending on infection stage [77,78]. However, when analysing the four subgroups of pigs, the differences were no longer significant, which can be explained by the smaller sample size per group and the consequent reduction in statistical power.

## 5. Conclusions

The present study demonstrated that Cirbloc^®^ M Hyo vaccination conferred virological protection in pigs subclinically infected with PCV2, as well as in those with high MDAs at the time of vaccination. These findings support the use of PCV2d genotype-based vaccines in piglets with varying MDA levels to promote effective viral heterologous control.

## Figures and Tables

**Table 1:** Comparison of the mean values of body weight (BW) and average daily weight gain (ADWG) at different time points between V and NV and among the 4 subgroups. Values include standard deviation and the coefficient of variation, in percentage (in brackets). Different superscript letters indicate statistically significant differences between groups for each time point (*p* < 0.05). wpv: weeks post-vaccination; wpc: weeks post-challenge; H: high; L: low; V: vaccinated; NV: not vaccinated.

		BW (Kg) $\overset{—}{X}$± SD (CV%)			ADWG (Kg) $\overset{—}{X}$ ± SD (CV%)		
Group		Vaccination	Challenge (5 wpv)	Necropsy (3 wpc)	(Vaccination– Challenge)	(Challenge– Necropsy)	(Vaccination–Necropsy)
**V vs. NV**	**V**	5.72 ± 0.96 (16.8%)	23.80 ± 4.42 (18.6%)	42.70 ± 6.39 (15.0%)	0.463 ± 0.096 (20.7%)	0.840 ± 0.110 (13.0%)	0.602 ± 0.092 (15.3%)
	**NV**	5.58 ± 1.07 (19.1%)	23.90 ± 3.80 (15.9%)	43.00 ± 6.47 (15.0%)	0.469 ± 0.084 (17.9%)	0.851 ± 0.154 (18.1%)	0.609 ± 0.098 (16.1%)
**All subgroups**	**H-V**	5.91 ± 1.02 (17.3%)	24.81 ± 3.28 (13.2%)	44.45 ± 4.18 (9.4%)	0.485 ± 0.065 (13.4%)	0.873 ± 0.081 ^a,b^ (9.2%)	0.627 ± 0.653 (8.9%)
	**H-NV**	5.63 ± 1.08 (19.2%)	24.45 ± 3.73 (15.2%)	45.77 ± 5.49 (12.0%)	0.482 ± 0.090 (18.6%)	0.947 ± 0.116 ^a^ (12.3%)	0.653 ± 0.088 (13.4%)
	**L-V**	5.52 ± 0.89 (16.2%)	22.69 ± 5.33 (23.5%)	40.84 ± 7.94 (19.4%)	0.440 ± 0.120 (27.2%)	0.805 ± 0.129 ^a,b^ (16.0%)	0.575 ± 0.035 (20.4%)
	**L-NV**	5.55 ± 1.10 (19.8%)	23.46 ± 3.96 (16.9%)	40.96 ± 6.58 (16.1%)	0.459 ± 0.082 (17.9%)	0.779 ± 0.142 ^b^ (18.2%)	0.576 ± 0.095 (16.5%)

**Table 2:** ISH results obtained in TBLNs for V vs. NV groups, as well as for all subgroups. The results are expressed as the number (%) of animals that received each score (0–3). Different superscript black letters indicate statistically significant differences between groups for each score (*p* < 0.05), while blue letters indicate a trend towards statistical significance (*p* < 0.1) between groups for each score. ISH: in situ hybridisation; NV: not vaccinated; V: vaccinated; H: high; L: low.

ISH (*n*/Total Pigs Per Group, %)					
Score (0–3)		0	1	2	3
**V vs. NV**	**V**	17/23 ^a^ (73.9%)	3/23 ^a^ (13.0%)	3/23 ^a^ (13.0%)	0/23 ^a^ (0.0%)
	**NV**	7/21^b^ (33.3%)	3/21 ^a^ (14.3%)	7/21 ^a^ (33.3%)	4/21 ^b^ (19.0%)
**All subgroups**	**H-V**	8/12 ^a^ (66.7%)	3/12 ^a^ (25.0%)	1/12 ^a^ (8.3%)	0/12 ^a^ (0.0%)
	**H-NV**	1/9 ^b^ (11.1%)	2/9 ^a^ (22.2%)	3/9 ^a^ (33.3%)	3/9 ^b^ (33.3%)
	**L-V**	9/11 ^a^ (81.8%)	0/11 ^a^ (0.0%)	2/11 ^a^ (18.2%)	0/11 ^a^ (0.0%)
	**L-NV**	6/12 ^a,b^ (50.0%)	1/12 ^a^ (8.3%)	4/12 ^a^ (33.3%)	1/12 ^a,b^ (8.3%)

## Data Availability

Data are contained within the article.
